# Relationship between individual alpha peak frequency and attentional performance in a multiple object tracking task among ice-hockey players

**DOI:** 10.1371/journal.pone.0251443

**Published:** 2021-05-27

**Authors:** Yanhui Zhang, Yingzhi Lu, Dandan Wang, Chenglin Zhou, Chang Xu

**Affiliations:** 1 School of Psychology, Shanghai University of Sport, Shanghai, China; 2 School of Kinesiology, Harbin Sport University, Harbin, China; Nottingham Trent University, UNITED KINGDOM

## Abstract

Individual alpha peak frequency (IAPF), the discrete frequency with the highest power value in the alpha oscillation range of the electroencephalogram, is a stable neurophysiological marker and is closely associated with various cognitive functions, including aspects of attention and working memory. However, the relationship between IAPF and attentional performance as well as the effects of engaging attention on IAPF are unknow. Here, we examined whether IAPF values were associated with attentional performance by evaluating accuracy during the performance of a multiple object tracking (MOT) task, a well-established paradigm for investigating goal-driven attention in dynamic environments, and whether engagement in the task affected IAPF values. In total, 18 elite players and 20 intermediate players completed the study. Resting electroencephalogram recordings were obtained for 120 s while players kept their eyes open and an additional 120 s while players’ eyes were closed, before and again after performing the MOT task. Tracking accuracy in the MOT task and IAPF values before and after the MOT task were analyzed. As expected, tracking accuracies were higher in elite players than in intermediate-level players. Baseline IAPF values were significantly and positively correlated with the accuracy of target tracking in the MOT task. IAPF values were higher in elite than intermediate players in both the eyes open and closed conditions and both before and after MOT task performance. Interindividual IAPF values did not differ before and after the MOT task. These findings indicate that IAPF is a stable marker, without intraindividual changes associated with engagement in the MOT task. Elite players had higher IAPF values and exhibited more accurate MOT performance than intermediate-level players; thus, baseline IAPF values may be useful to predict attentional performance in the MOT task among athletes.

## Introduction

For several decades, electroencephalography (EEG) has been used substantially in neuroscientific research. Brain activity is reflected by five prominent EEG biomarkers defined by their frequency bands: the delta-band (<4 Hz), theta-band (4–7 Hz), alpha-band (7.5–12.5 Hz), beta-band (13–30 Hz), and gamma-band (30–70 Hz). These different EEG bands represent individual neural states associated with different types of behavioral processing (e.g., sleep, relaxation, alertness, and attention). In humans, the alpha-band rhythm is most prominently observed over the occipital and parietal cortices [1] and may reflect cognitive performance [2,3], selective attention [4], inhibition, and gating [5]. The amplitude of the alpha-band oscillation is at its peak in an eyes-closed resting condition, primarily due to lack of sensory input. Suppression of the amplitude of the alpha-band oscillation occurs in response to the eyes opening.

Individual alpha peak frequency (IAPF) is the discrete frequency with the highest power value in the alpha oscillation range (7.5–12.5 Hz) [6]. IAPF has been shown to be a stable neurophysiological marker [7]. A large study by Grandy et al. [8] affirmed the stability of IAPF as a neurophysiological marker by comparing pre- versus post-training IAPF values across groups of younger and older people, including healthy adults up to 80 years of age, and found that IAPF remained unaltered by cognitive interventions. Similarly, Christie et al. [9] observed no significant changes in IAPF when comparing baseline IAPF and pre- to post-task IAPF values across three performance levels. IAPF may also reflect traits of individuals with certain brain pathologies. A study by Angelakis et al. [7] found that individuals with traumatic brain injury had lower IAPFs than healthy individuals in a post-task eyes open (EO) resting condition, consistent with the suggestion that IAPF may reflect a cognitive preparedness trait. In a follow-up experiment, on examination of the cognitive preparedness state, the authors found that IAPF values of healthy participants in an eyes closed (EC) pre-task condition correlated with working memory performance on the first day of memory testing. Their results indicated that baseline IAPF can fluctuate within individuals and further suggested that IAPF may reflect moment-to-moment cognitive preparedness [7]. Thus, IAPF appears to be both a stable neurophysiological trait marker as well as a state variable with intraindividual variability.

IAPF has also been shown to correlate strongly with diverse cognitive performance variables, such as conflict reaction-time speed [10], anticipation [11], and sentence-processing speed [12]. A study by Bornkessel et al. [12] found that interindividual differences in language comprehension were detected only when participants were classified according to IAPF, whereas classifications based on reading span, processing speed, and processing accuracy failed to yield reliable group differences. They found that individuals with lower IAPFs show higher processing loads when processing ambiguous portions of sentences while reading than individuals with higher IAPFs. Similarly, a study by Rathee et al. [13] found that individuals with high IAPFs performed better on a reading comprehension task than individuals with low IAPFs, suggesting that IAPF may provide a suitable neurophysiological means of categorizing interindividual differences in language processing. In a study examining the association between IAPF and memory, Klimesch et al. [14] found that IAPF correlated with memory task performance, suggesting that IAPF may be an index of memory ability. Similarly, the study by Angelakis et al. [7] showed that IAPFs in healthy individuals correlated with working memory performance. These experimental results suggest that IAPF may be a predictor of cognitive performance in a working memory task performed close in time to the EEG recording. A study by Jann et al. [15] showed that individuals with higher baseline IAPFs had lower neural activation (as assessed by blood-oxygen-level-dependent signals) in response to stimuli and hypothesized that a high pre-task IAPF may reflect pre-activated task-relevant networks and thus yield greater efficiency in task implementation.

It has been suggested that shifts in IAPF are detected only when a strong effort is being made involving cardiovascular and metabolic processes [16]. A study by Hülsdünker et al. [17] found that a positive shift in IAPF was associated with increasingly difficult balancing tasks, suggesting that the shift was related to an “increase in cortical resource investment and activation.” Gutmann et al. [18] found that IAPF increased after exhaustive exercise, but not after steady-state exercise; they further showed that augmented IAPF values remain elevated for less than 30 minutes after cessation of exhaustive exercise [19]. IAPF values also change in response to engagement in a cognitive task. Haegens et al. [20] observed an increase in IAPF when individuals transitioned from a resting state to a passive visual stimulation condition in the n-back paradigm. Their n-back paradigm consisted of a 0-back and a 2-back task presented in an alternating manner. In the 0-back task, participants responded by pressing a button whenever the letter X appeared; in the 2-back task, participants responded when the stimulus was the same as that presented two stimuli back. Their study found that participants showed a significantly higher IAPF in the 2-back condition than in the 0-back condition. Because IAPF increases during cognitive, memory, and sensorimotor task performances as well as in response to a strenuous bout of physical exercise, these increases may reflect the activation of different groups of neurons [21] or may reflect active engagement of a system associated with increased cognitive demand [9]. In addition, it is unclear why IAPF is resistant to change in some paradigms but not others [9]. Additional research is needed to elucidate the neurological mechanisms underlying IAPF shifts.

Although IAPF has been correlated with various forms of cognitive performance, there are limited data regarding the relationship between IAPF and attention, with the notable exception of studies reporting that individuals with attention-deficit/hyperactivity disorder have lower IAPFs than those typically detected among individuals without the disorder [22]. The relationship between IAPF and attentional performance, as measured with an attention task, and the effects of engaging attention on IAPF are unknown. Therefore, the aim of the present study was to fill in these gaps in the literature. Attentional performance was assessed using a multiple object tracking (MOT) task, a well-established paradigm for investigating goal-driven attention in dynamic environments. Ice-hockey players were selected for this study because attention plays an important role in the game of ice-hockey. Players need to track the puck while monitoring other players’ positions and movements on the ice and use this dynamic information to perform the higher-order cognitive task of making tactical play decisions, such as whether to pass or shoot the puck, all while themselves making fast movements and frequent directional changes on the ice [23]. Elite athletes have been shown to exhibit better tracking of multiple objects than novices [24] and also to track objects better than both non-athletes and intermediate athletes under high-difficulty task conditions [25]. Given those findings, we hypothesized that (a) elite-level ice-hockey players would exhibit higher levels of attentional performance than intermediate-level players in the MOT task; (b) baseline IAPFs would correlate with MOT task performance; and (c) IAPF values would be stable from pre- to post-MOT task.

## Materials and methods

### Participants

Thirty-eight players (18 women, 20 men) who were members of the Harbin Sport University ice-hockey teams participated in this study, including 18 elite-level players (mean age, 20.65 ± 2.61 years; range, 18–25 years; 10 men and 8 women) and 20 intermediate-level players (mean age, 20.5 ± 3.14 years; range, 18–24 years; 10 men and 10 women). The elite players were Master Sportsman and national first-level athletes, whereas the intermediate players were national second-level players and physical education students majoring in ice-hockey at the university. All participants were right-handed and reported normal or corrected-to-normal vision. They were asked to avoid staying up late before participating in the study to ensure good quality of sleep, and to avoid vigorous exercise to ensure good physical condition for the experiment. The experimental protocol was approved by the regional ethics committee of Shanghai University of Sport (No. 102772019RT011) and was conducted according to the principles expressed in the Declaration of Helsinki. All participants provided written informed consent prior to the start of the experiment. Five individuals (3 from the elite group and 2 from the intermediate group) were excluded from the final analysis due to faulty baseline EEG recordings.

### Procedures

For baseline data collection, resting EEGs were recorded for 120 s with the player’s eyes closed (EC) followed by 120 s with their eyes open (EO). Immediately after the baseline EEG recording, each participant completed the MOT task. Immediately after completing the MOT task, a post-MOT resting EEG recording was completed, also with 120-s EC and EO periods. Findings by Angelakis et al. [7] suggest that *inter*individual variability is best observed during pre-task rest periods and that IAPF differences between pre- and post-task rest periods reflect *intra*individual variability. Therefore, in the present study, IAPF was calculated at two time points for each individual to determine *inter*individual and *intra*individual variability relative to the MOT task.

### Attention assessment

**Fig 1:**
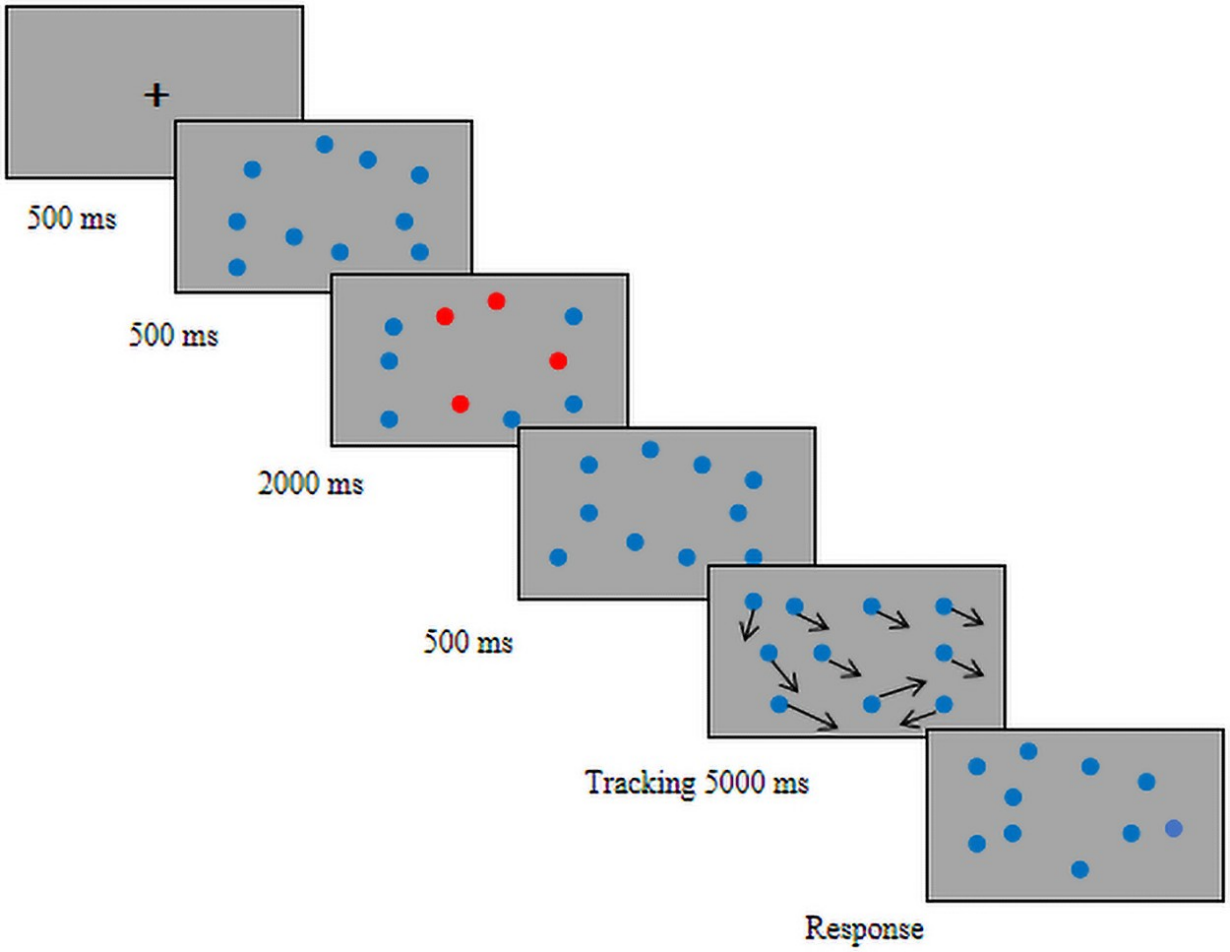
Schematic diagram of the multiple object tracking task. Ten identical blue objects are presented, and four target objects turn red for 2000 ms before turning back to blue. After 1500 ms, all objects move at a speed of 5°/s for 5000 ms. At the end of the tracking period, the objects stop moving, and participants select the four targets by clicking on each one using the left mouse button.

Pylyshyn and Storm [26] originally developed the MOT task to explore dynamic, spatiotemporal attention. The MOT task has been used to assess several aspects of attention, including selective, distributed, and sustained attention [27]. The MOT task in the present study was conducted on a 2.5-GHz Dell desktop computer running Windows 7. Visual stimuli were created in MATLAB and presented on a 23.8-inch LCD monitor (resolution, 1920 × 1080 pixels; refresh rate, 60 Hz). The experiment was conducted in a quiet, lit room. Participants, seated 57 cm from the screen, responded to stimuli using a mouse. For each trial, a black fixation point was first presented for 500 ms on a gray background (25° × 17°), followed by 10 identical blue objects (diameter, 1 cm) for 500 ms. Subsequently, four of the objects were highlighted by turning red for 2000 ms to designate them as tracking targets. The targets then reverted to blue so that no cue remained to distinguish them from non-target items. After 500 ms, all 10 objects moved in random directions at a constant speed (5°/s) for 5000 ms. At the end of the tracking period, the objects stopped moving. The participants were instructed to click the left mouse button to select the four targets. The participants needed to click four times in each trial, once on each target, and they advanced to the next trial by pressing the keyboard space bar (Fig 1). Initial object positions were generated randomly from trial to trial. Objects made random changes every second to make their movements unpredictable. To avoid collision or overlap, the objects were programmed to change direction when approaching one another or touching the screen border. Participants completed a total of 20 trials.

### EEG data acquisition

EEG activity was recorded from 64 Ag/AgCl electrodes arranged according to the International 10–20 System at a sampling rate of 1000 Hz via a BrainVision Recorder (version 2.0, Brain Products GmbH; Munich, Germany). Participants were instructed to sit relaxed during EEG recordings. EEG recordings were referenced online against the FCz site and were grounded at the AFz site. The vertical and horizontal electrooculograms were placed below the left eye and lateral to the right eye, respectively. Two electrodes were placed on the left and right mastoids for offline re-referencing. Electrode impedances were kept <10 kΩ for the duration of the experiment. Resting-state EEG data consisted of pre- and post-MOT task EEG recordings, assessed during the 120-s EC and EO periods.

### EEG data analysis

EEG data were processed in BrainVision Analyzer 2 (version 2.1, Brain Products GmbH). The signals were re-referenced relative to the mean left and right mastoid signals [28]. The epochs were band-pass filtered with a low cutoff of 0.1 Hz and a high cutoff of 30 Hz (slope, 24 dB/oct). An independent component analysis [29] was conducted to correct for vertical and horizontal eye movements. The ocular artifact–corrected EEG data were segmented into 1000-ms epochs. The data were processed using a semi-artifact rejection method, with the vertical and horizontal electrooculogram channels excluded from the artifact search. Segments with amplitudes exceeding −80 μV or 80 μV were rejected. The data were subjected to fast Fourier transform, with a Hanning window yielding a 0.1-Hz frequency resolution. On the basis of relevant published research and the topography of our own data, IAPF identification was conducted by exploring source-level power spectra at two occipital (O1 and O2) and five parietal (P7, P3, Pz, P4, and P8) sites [9,30].

### Statistical analysis

Statistical analyses were completed in SPSS, version 24.0 (SPSS Inc., Chicago, IL). For the behavioral data, we calculated the percentage of correct responses (accuracy), that is, the number of correctly selected targets out of the total target number. Tracking performance was compared between the elite and intermediate groups with independent samples *t* tests.

For the EEG data, IAPFs were estimated as the mean of the peak alpha frequency, which was obtained by visual inspection, over O1, O2, P7, P3, Pz, P4, and P8 electrode sites. IAPF was estimated separately for the EC and EO conditions. Correlations between IAPF in the four conditions (pre- and post-MOT task with EC and EO) and MOT task performance were evaluated with Pearson correlation analyses. IAPF shifts across the pre- and post-MOT EO conditions were analyzed with a two-way mixed analysis of variance (ANOVA), with one between-subjects factor (group: elite vs. intermediate players) and one within-subject factor (time: pre- vs. post-MOT task). IAPF was also compared between the elite and intermediate-level players across all four conditions (pre- and post-MOT task with EC and EO) using independent samples *t* tests. An alpha level of 0.05 was pre-selected for all statistical comparisons. Effect sizes were classified as small (0.10), medium (0.25), and high (0.40). Among 33 participants, the 95% confidence intervals (CIs) for IAPFs pre-MOT task were 9.46–10.39 Hz for EO and 9.88–10.58 Hz for EC, and post-MOT task they were 9.52–10.30 Hz for EO and 9.80–10.46 Hz for EC. The 95% CI for accuracy was 0.58–0.64 (proportion of) correct responses.

## Results

### Attention assessed in the MOT task

**Fig 2:**
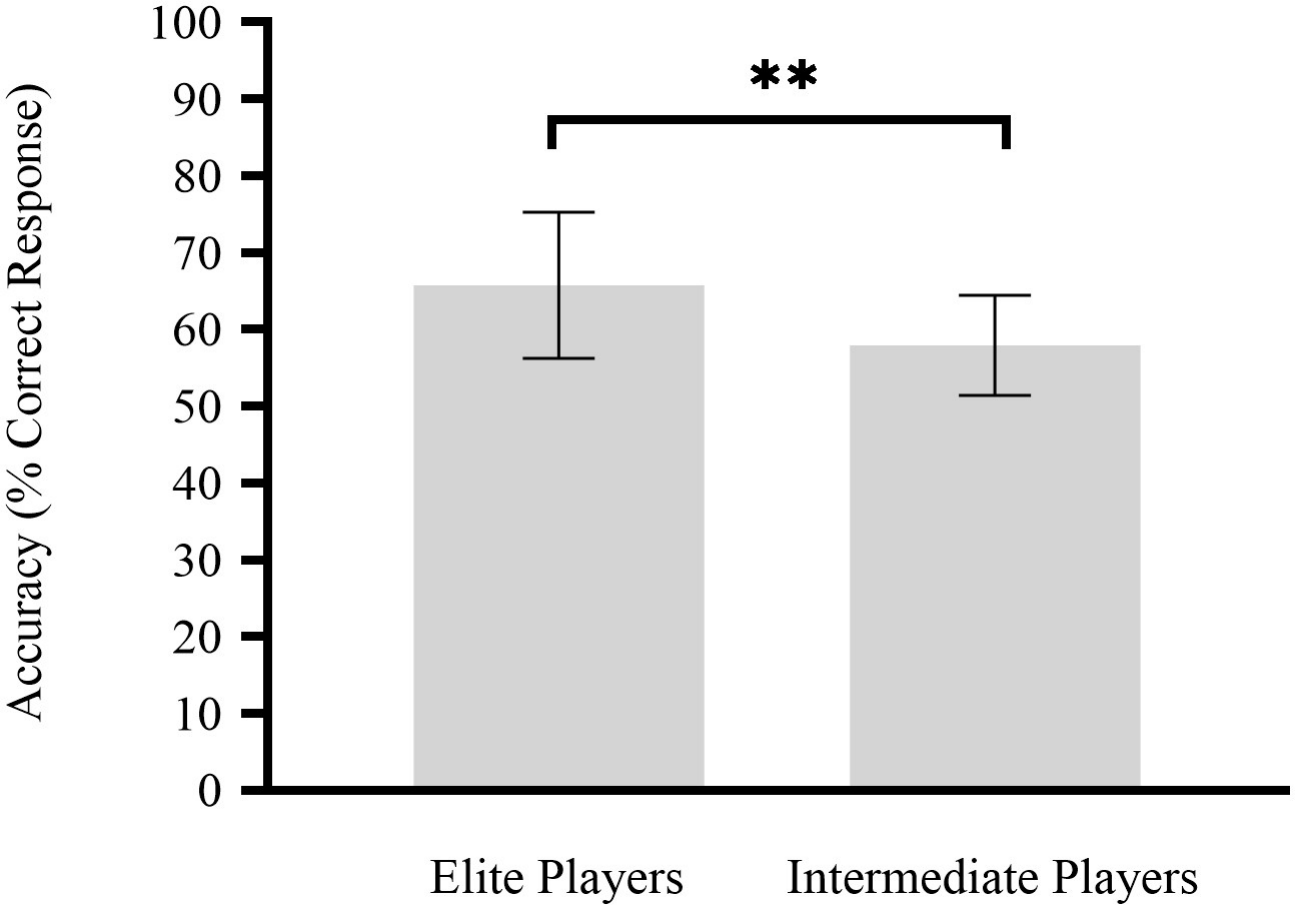
Attention as assessed in ice-hockey players performing the multiple object tracking task. ** *p*<0.01.

Overall, participant tracking accuracy in the MOT task was 61.49% ± 8.82%. As shown in Fig 2, tracking accuracy among 15 elite ice-hockey players (mean [standard deviation] correct responses, 65.75% [9.51%]) was significantly better (*t*_1,31_ = 2.79, *p* < 0.01) than that among 18 intermediate players (mean [standard deviation] correct responses, 57.94% [6.51%]).

### Correlations between IAPF and tracking accuracy in the MOT task

As shown in Table 1, IAPFs for the pre-MOT task were significantly and positively correlated with accuracy in both groups for the EC condition, with elite players exhibiting a more robust correlation than intermediate players, whereas nonsignificant trends for negative correlations with tracking accuracy in both groups were detected for the EO condition. The IAPFs for the post-MOT task also showed only nonsignificant trends for correlations with accuracy in both groups of athletes.

**Table 1:** Correlations between IAPFs and tracking accuracy in the MOT task, stratified by eyes open (EO) or closed (EC) and by player group.

Player Group	Pearson *r*			
	Pre-MOT		Post-MOT	
	EO	EC	EO	EC
Elite (n = 15)	-0.35	0.52*	-0.06	-0.21
Intermediate (n = 18)	-0.18	0.48*	-0.18	0.21

Abbreviations. IAPF, individual alpha peak frequency; MOT, multiple object tracking.

*p < 0.05.

### Change in IAPF related to MOT task engagement

**Fig 3:**
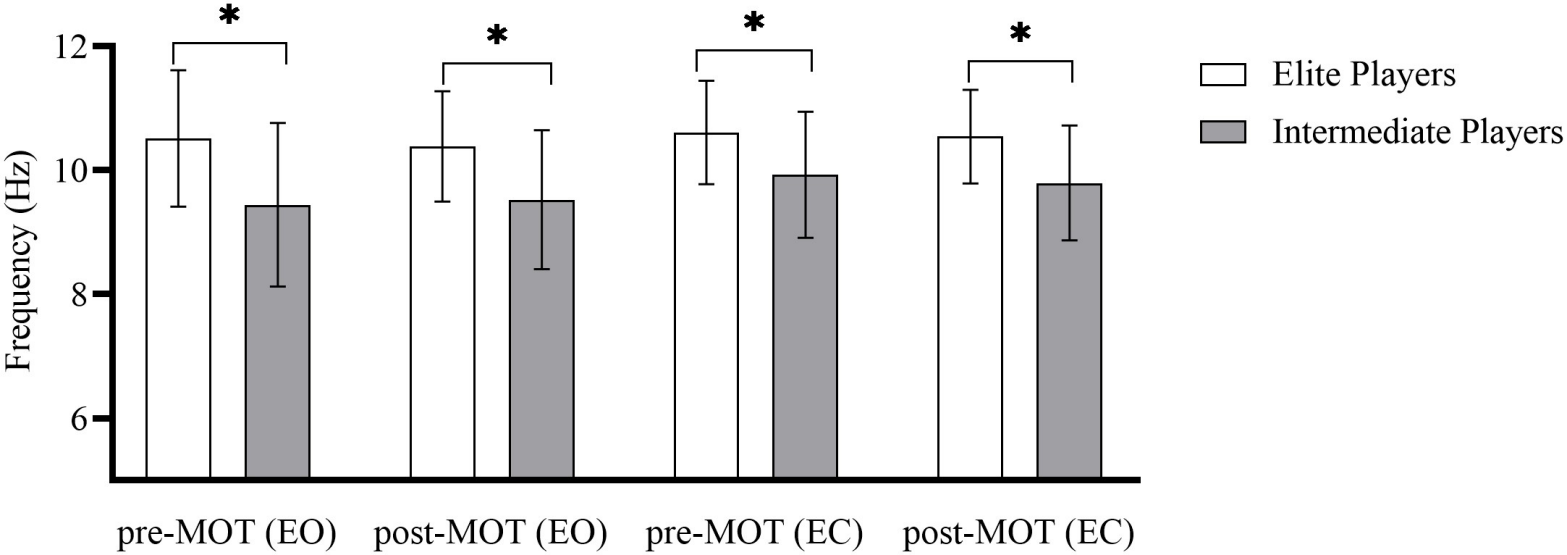
Changes in individual alpha peak frequency associated with MOT task engagement. Data bars are means, and error bars are standard deviation. MOT represents multiple object tracking; EC, eyes closed; and EO, eyes open; *p < 0.05.

The results of a 2 × 2 (elite vs. intermediate × pre- vs. post-MOT) mixed ANOVA for IAPF in the EO condition indicated a significant main effect of group (*F*_(1,31)_ = 8.01, *p* < 0.01, η_p_^2^ = 0.21). Post hoc analyses indicated that IAPFs in the pre- and post-MOT task EO conditions were significantly higher among the elite players than among the intermediate players (Fig 3). There was not a main effect of time (*F*_(1,31)_ = 0.29, *p* > 0.05) nor a significant time by group interaction (*F*_(1,31)_ = 0.03, *p* > 0.05).

Similarly, a 2 × 2 (elite vs. intermediate × pre- vs. post-MOT) mixed ANOVA for IAPF in the EC condition revealed a significant main effect of group (*F*_(1,31)_ = 7.91, *p* < 0.01, η_p_^2^ = 0.20). Post hoc analyses indicated that for the EC condition, IAPFs in the pre- and post-MOT tasks were significantly higher among the elite players than among the intermediate players (Fig 3) There was not a significant main effect of time (*F*_(1,31)_ = 0.29, *p* > 0.05) nor a significant time by group interaction (*F*_(1,31)_ = 0.0115, *p* > 0.05).

## Discussion

In the present study, we found that baseline IAPFs were positively correlated with the accuracy of tracking performance in the MOT task, without significant *intra*individual IAPF changes associated with engagement in the MOT task (i.e., pre- vs. post-MOT task). The tracking accuracies of the elite ice-hockey players were significantly better than those of the intermediate-level players.

On the basis of the relevant literature, we believe that the MOT task is a suitable measure for comparing attentional performance across individuals with different levels of ice-hockey play mastery because the task resembles, to some extent, the puck and player tracking processes required of ice-hockey players on the ice in the real world [27]. Our observation of superior target tracking by elite players is consistent with prior evidence regarding faster attentive visual tracking of multiple moving objects by athletes who play ball-involved team sports [23]. Thus, our results extend those of previous studies and confirm our expectation that expertise in team ball sports transfers to the MOT task.

IAPF values have been previously associated with various types of cognitive performance. A study by Bazanova and Aftanas [31] found a positive association between performance accuracy on the Torrance test (fluency subfactor) and maximum peak frequency. Another study by Grandy et al. [30] concluded that IAPF values are associated with latent general cognitive factors. Similarly, in the present study, we observed a significant positive correlation between baseline IAPFs and the players’ tracking performance levels. Our findings of higher IAPFs in elite players than in intermediate-level players in the pre-MOT EC condition indicate that such a baseline IAPF measure may predict the ability of ice-hockey players to perform tasks that involve selective, distributed, and sustained attention, with a higher baseline IAPF being associated with both MOT task performance and ice-hockey mastery.

Furthermore, IAPF values have been previously shown to positively correlate with memory task performance [7] and negatively correlate with the speed of conflict perception [10]. Thus, baseline IAPF can predict the speed of visual reaction and working memory performance among non-athletes, as well as the target tracking performance of ice-hockey players. We showed in the present study that baseline IAPF may be considered a putative index of attentional processing ability, with a higher baseline IAPF being associated with a better preparation status and better task execution. Our findings confirmed our hypothesis that elite ice-hockey players would have high baseline IAPF values. Based on these findings, we further hypothesize that this elevated IAPF may reflect attention-relevant network states and thus enable more efficient task execution. If so, baseline IAPF could potentially be used to help identify elite athletes. Seemingly contrary to our results, Angelakis and colleagues found that IAPF values recorded during and after reading, but not before (rest baseline), correlated with verbal and attentional cognitive trait abilities in young adults [32]. The authors suggested that IAPF may reflect some general cognitive abilities, such as cognitive preparedness, response control, or the ability to acquire vocabulary. If so, cognitive traits may reflect the ability to induce cognitive states. Similarly, in the sensorimotor domain, intratask IAPF was previously found to be associated with the level of balance control demand [17]. A prior study examining the relationship of baseline IAPF and performance in an ice-hockey puck shooting task did not show differences across three performance levels of the participants [9], perhaps because all of the participants were university athletes with ability levels that were not substantially differentiated (their “levels” were based on task performance scores). In the present study, we compared more pronounced ability-differentiated athlete groups and found that sport mastery was associated with both higher baseline IAPF and more efficient task execution, perhaps owing to more ready attentional networks at rest. Future research could assess IAPF during the MOT task to explore this postulation.

Our findings of non-significant differences in IAPFs across pre- and post-MOT EO and EC conditions for both elite and intermediate-level ice-hockey players indicated that IAPF is stable and not readily amenable to change by MOT task performance. These findings are consistent with previous studies concluding that IAPF is a stable neurophysiological marker [7] and that it is not altered by engagement in a cognitive task, even an attention-related one. It appears that IAPF shifts may occur only when an individual exerts a strong effort involving cardiovascular and metabolic processes, and thus such shifts could reflect mental or physical fatigue [16]. In this context, Gutmann et al. [18] detected a significant increase in IAPF after exhaustive exercise but not after steady-state exercise, suggesting an IAPF increase may reflect a heightened level of preparedness for external input or heightened arousal that could facilitate information processing.

Given these findings, it is plausible that the MOT task may not have been sufficiently physically or cognitively demanding to affect IAPF. Some minor fatigue after finishing the MOT task may be reflected by decreased IAPF values in the EC condition, whereas a need to correct for unpreparedness for the task may be reflected by a slight increase. Our findings of generally higher IAPFs in elite players than intermediate-level players across the four conditions may reflect higher arousal, which may facilitate information processing.

The present study has a few limitations that should be taken into consideration. First, we did not measure IAPFs during task performance, but rather before and after. Second, the analysis did not take into account the players’ sex, which may have influenced the findings.

## Conclusions

Baseline IAPFs of ice-hockey players were correlated with attentional performance in the MOT task, with elite players exhibiting higher IAPF values and better tracking performance (accuracy) than intermediate-level players. The IAPF values remained stable within individuals before versus after performance of the MOT task. Our findings indicated that IAPF values reflected high levels of attention in ice-hockey players performing the MOT task and suggest that these values may be useful for selecting elite athletes: the higher the IAPF values and level of attention (as reflected in greater accuracy in the MOT task), the higher the level of the athlete. Future research should explore correlations of baseline IAPF (ensuring proper sports-specific mental preparation) during and after sports performance.

## Supporting information

S1 File Raw tracking accuracy data. (XLSX)

## References

[1] Cheron G, Petit G, Cheron J, Leroy A, Cebolla A, Cevallos C, et al. Brain Oscillations in Sport: Toward EEG Biomarkers of Performance. Front Psychol. 2016; 7:246. 10.3389/fpsyg.2016.00246 . 26955362 PMC4768321

[2] Klimesch W, Doppelmayr M, Schimke H, Pachinger T. Alpha frequency, reaction time, and the speed of processing information. J Clin Neurophysiol. 1996; 13:511–8. 10.1097/00004691-199611000-00006 . 8978623

[3] Richard Clark C, Veltmeyer MD, Hamilton RJ, Simms E, Paul R, Hermens D, et al. Spontaneous alpha peak frequency predicts working memory performance across the age span. Int J Psychophysiol. 2004; 53:1–9. 10.1016/j.ijpsycho.2003.12.011 . 15172130

[4] Mulholland T. The concept of attention and the EEG alpha-rhythm. Electroencephalogr Clin Neurophysiol. 1968; 24:188. 4170491

[5] Jensen O, Mazaheri A. Shaping functional architecture by oscillatory alpha activity: gating by inhibition. Front Hum Neurosci. 2010; 4:186. 10.3389/fnhum.2010.00186 . 21119777 PMC2990626

[6] Klimesch W. EEG alpha and theta oscillations reflect cognitive and memory performance: a review and analysis. Brain Research Reviews. 1999; 29:169–95. 10.1016/s0165-0173(98)00056-3 . 10209231

[7] Angelakis E, Lubar JF, Stathopoulou S, Kounios J. Peak alpha frequency: an electroencephalographic measure of cognitive preparedness. Clin Neurophysiol. 2004a; 115:887–97. 10.1016/j.clinph.2003.11.034 . 15003770

[8] Grandy TH, Werkle-Bergner M, Chicherio C, Schmiedek F, Lövdén M, Lindenberger U. Peak individual alpha frequency qualifies as a stable neurophysiological trait marker in healthy younger and older adults. Psychophysiology. 2013a; 50:570–82. 10.1111/psyp.12043 . 23551082

[9] Christie S, Di Fronso S, Bertollo M, Werthner P. Individual Alpha Peak Frequency in Ice Hockey Shooting Performance. Front Psychol. 2017; 8:762. 10.3389/fpsyg.2017.00762 . 28559868 PMC5433296

[10] Jin Y, O’Halloran JP, Plon L, Sandman CA, Potkin SG. Alpha EEG predicts visual reaction time. Int J Neurosci. 2006; 116:1035–44. 10.1080/00207450600553232 . 16861166

[11] Basar E, Yordanova J, Kolev V, Basar-Eroglu C. Is the alpha rhythm a control parameter for brain responses. Biological Cybernetics. 1997; 76:471–80. 10.1007/s004220050360 . 9263433

[12] Bornkessel ID, Fiebach CJ, Friederici AD, Schlesewsky M. "Capacity" reconsidered: interindividual differences in language comprehension and individual alpha frequency. Exp Psychol. 2004; 51:279–89. 10.1027/1618-3169.51.4.279 . 15620229

[13] Rathee S, Bhatia D, Punia V, Singh R. Peak Alpha Frequency in Relation to Cognitive Performance. J Neurosci Rural Pract. 2020; 11:416–9. 10.1055/s-0040-1712585 . 32753806 PMC7394609

[14] Klimesch W, Schimke H, Pfurtscheller G. Alpha frequency, cognitive load and memory performance. Brain Topography. 1993; 5:241–51. 10.1007/BF01128991 . 8507550

[15] Jann K, Koenig T, Dierks T, Boesch C, Federspiel A. Association of individual resting state EEG alpha frequency and cerebral blood flow. Neuroimage. 2010; 51:365–72. 10.1016/j.neuroimage.2010.02.024 . 20156573

[16] Billiot KM, Budzynski TH, Andrasik F. EEG Patterns and Chronic Fatigue Syndrome. Journal of Neurotherapy. 1997; 2:20–30. 10.1300/J184v02n02_04

[17] Hülsdünker T, Mierau A, Strüder HK. Higher Balance Task Demands are Associated with an Increase in Individual Alpha Peak Frequency. Front Hum Neurosci. 2015; 9:695. 10.3389/fnhum.2015.00695 . 26779005 PMC4702132

[18] Gutmann B, Mierau A, Hülsdünker T, Hildebrand C, Przyklenk A, Hollmann W, et al. Effects of physical exercise on individual resting state EEG alpha peak frequency. Neural Plast. 2015; 2015:1–6. 10.1155/2015/717312 . 25759762 PMC4338399

[19] Gutmann B, Zimmer P, Hülsdünker T, Lefebvre J, Binnebößel S, Oberste M, et al. The effects of exercise intensity and post-exercise recovery time on cortical activation as revealed by EEG alpha peak frequency. Neurosci Lett. 2018; 668:159–63. 10.1016/j.neulet.2018.01.007 . 29329910

[20] Haegens S, Cousijn H, Wallis G, Harrison PJ, Nobre AC. Inter- and intra-individual variability in alpha peak frequency. Neuroimage. 2014; 92:46–55. 10.1016/j.neuroimage.2014.01.049 . 24508648 PMC4013551

[21] Sadaghiani S, Scheeringa R, Lehongre K, Morillon B, Giraud A-L, Kleinschmidt A. Intrinsic connectivity networks, alpha oscillations, and tonic alertness: a simultaneous electroencephalography/functional magnetic resonance imaging study. J Neurosci. 2010; 30:10243–50. 10.1523/JNEUROSCI.1004-10.2010 . 20668207 PMC6633365

[22] Lansbergen MM, Arns M, van Dongen-Boomsma M, Spronk D, Buitelaar JK. The increase in theta/beta ratio on resting-state EEG in boys with attention-deficit/hyperactivity disorder is mediated by slow alpha peak frequency. Prog Neuropsychopharmacol Biol Psychiatry. 2011; 35:47–52. 10.1016/j.pnpbp.2010.08.004 . 20713113

[23] Faubert J. Professional athletes have extraordinary skills for rapidly learning complex and neutral dynamic visual scenes. Sci Rep. 2013; 3:1154. 10.1038/srep01154 . 23378899 PMC3560394

[24] Mangine GT, Hoffman JR, Wells AJ, Gonzalez AM, Rogowski JP, Townsend JR, et al. Visual tracking speed is related to basketball-specific measures of performance in NBA players. Journal of Strength and Conditioning Research. 2014; 28:2406–14. 10.1519/JSC.0000000000000550 . 24875429

[25] Qiu F, Pi Y, Liu K, Li X, Zhang J, Wu Y. Influence of sports expertise level on attention in multiple object tracking. PeerJ. 2018; 6:e5732. 10.7717/peerj.5732 . 30280051 PMC6166630

[26] Pylyshyn ZW, Storm RW. Tracking multiple independent targets: evidence for a parallel tracking mechanism. Spat Vis. 1988; 3:179–97. 10.1163/156856888x00122 . 3153671

[27] Faubert J, Sidebottom L. Perceptual-cognitive training of athlete. Journal of Clinical Sport Psychology. 2012; 6:85–102.

[28] Gootjes L, Coppens LC, Zwaan RA, Franken IHA, van Strien JW. Effects of recent word exposure on emotion-word Stroop interference: an ERP study. Int J Psychophysiol. 2011; 79:356–63. 10.1016/j.ijpsycho.2010.12.003 . 21156188

[29] Bell AJ, Sejnowski TJ. An information-maximization approach to blind separation and blind deconvolution. Neural Computation. 1995; 7:1129–59. 10.1162/neco.1995.7.6.1129 . 7584893

[30] Grandy TH, Werkle-Bergner M, Chicherio C, Lövdén M, Schmiedek F, Lindenberger U. Individual alpha peak frequency is related to latent factors of general cognitive abilities. Neuroimage. 2013b; 79:10–8. 10.1016/j.neuroimage.2013.04.059 . 23624490

[31] Bazanova OM, Aftanas LI. Individual measures of electroencephalogram alpha activity and non-verbal creativity. Neuroscience and Behavioral Physiology. 2008; 38(3):227–35. 10.1007/s11055-008-0034-y . 18264769

[32] Angelakis E, Lubar JF, Stathopoulou S. Electroencephalographic peak alpha frequency correlates of cognitive traits. Neurosci Lett. 2004b; 371:60–3. 10.1016/j.neulet.2004.08.041 . 15500967

